# Compositional Analysis of the Associations between 24-h Movement Behaviours and Health Indicators among Adults and Older Adults from the Canadian Health Measure Survey

**DOI:** 10.3390/ijerph15081779

**Published:** 2018-08-18

**Authors:** Duncan E. McGregor, Valerie Carson, Javier Palarea-Albaladejo, Philippa M. Dall, Mark S. Tremblay, Sebastien F. M. Chastin

**Affiliations:** 1 Institute for Applied Health Research, School of Health and Life Science, Glasgow Caledonian University, Cowcaddens Road, Glasgow G4 0BA, Scotland, UK; philippa.dall@gcu.ac.uk (P.M.D.); sebastien.chastin@gcu.ac.uk (S.F.M.C.); 2 Biomathematics and Statistics Scotland, JCMB, The King’s Buildings, Peter Guthrie Tait Road, Edinburgh EH9 3FD, Scotland, UK.; javier.palarea@bioss.ac.uk; 3 Faculty of Kinesiology, Sport, and Recreation, 1-151 Van Vliet Complex, University of Alberta, Edmonton, AB T6G 2H9, Canada; vlcarson@ualberta.ca; 4 Healthy Active Living and Obesity Research Group, Children’s Hospital of Eastern Ontario Research Institute, 401 Smyth Road, Ottawa, ON K1H 8L1, Canada; mtremblay@cheo.on.ca; 5 Department of Movement and Sport Science, Ghent University, 9000 Ghent, Belgium

**Keywords:** ageing, time use, sleep, physical activity, sedentary behaviour, compositional data analysis

## Abstract

This study investigated the association between the allocation of time-use over the 24-h day between sleep, sedentary behaviour (SB), light-intensity physical activity (LPA) and moderate-to-vigorous-intensity physical activity (MVPA)) and health indicators. A cross-sectional analysis of Canadian Health Measures Survey data was undertaken using compositional data analysis. SB, LPA and MVPA were derived from Actical accelerometers, whilst sleep was self-reported by respondents. The analysis was stratified by age; adults (aged 18–64 years; *n* = 6322) and older adults (65–79 years; *n* = 1454). For adults, beneficial associations were observed between larger proportions of MVPA relative to time in other behaviours and body mass index (BMI), waist circumference, aerobic fitness, resting heart rate, high-density lipoprotein (HDL) cholesterol, triglycerides, blood glucose and insulin levels. More time spent in sleep relative to other movement behaviours was deleteriously associated with aerobic fitness, HDL cholesterol, insulin, C-reactive proteins and grip strength but beneficially with low-density lipoprotein cholesterol. Relative time spent in LPA was deleteriously associated with BMI and beneficially with triglycerides and grip strength. In older adults, these associations were blunted or disappeared but larger proportions of MVPA were associated with better mental health. The importance to health of MVPA when explicitly considered relative to other movement behaviours was confirmed.

## 1. Introduction

It is widely accepted that physical inactivity is a major risk factor for non-communicable disease, disablement in later life and premature mortality [1,2,3]. It is therefore recommended to spend time daily engaging in moderate-to-vigorous-intensity physical activity (MVPA) at all ages [4]. However, over any 24-h period, movement occurs on a continuum from sleep (i.e., no/low movement) to vigorous-intensity physical activity (i.e., high movement). Evidence is now mounting that time spent in lower intensity daily movement behaviours are also associated with health and wellbeing [5]. Time spent sitting or in sedentary behaviour (SB) has been found to be detrimental to health [6]. On the contrary, time spent in light-intensity activity (LPA) incidental to daily living appears to have a positive effect on cardio metabolic health and mortality unless it displaces MVPA [7]. Finally, time spent sleeping is also associated with both deleterious and positive outcomes [8]. Alternative divisions are possible and a number of studies have gone further in considering the possibility that the longer periods of inactivity may have greater deleterious effects [9]. More recently, it was pointed out that none of these behaviours are really independent of each other. As there are 24 h in a day, trying to influence one of these behaviours would necessarily impact another. Rather than consider behaviours in isolation, it is more useful to consider overall time-use, that is how the time available to an individual in a day is allocated between different movement behaviours. Consequently, several studies have investigated the combined effect of 24-h movement behaviours on health using isotemporal and compositional data analysis techniques in the United States [5,10], Canada [11,12] and Australia [13]. Collectively, these studies showed that the whole 24-h time-use is associated with health indicators in children, adults and older adults and that it is important to understand synergistic effects among movement behaviours. Changing lifestyles are also driving new physical activity patterns, such as the well-known “weekend warrior” [14], leading to a need to better understand the combined effect of movement behaviours. At present, inactivity is a greater problem in high income countries, however it remains an issue for developing countries [15]. It is likely rapid economic development will alter this [16] and potentially give rise to new physical activity patterns.

Twenty-four-hour movement guidelines have now appeared for children aged 5–17 years in Canada [17] and for children aged 0-4 years in Canada, Australia and New Zealand [18,19,20]. Similar guidelines are very likely to emerge for older age groups in Canada [21], though the recommendations for these age groups might be different. In this study, we investigated the relationship between the composition of the 24-h movement behaviours with health indicators using the Canadian Health Measure Survey (CHMS) and compared findings between adults and older adults, who are more vulnerable to the health risks associated with the indicators considered. In particular, we considered which health indicators are associated with the composition of the 24-h day in adults, whether this was attributable to the relative level of MVPA, if the relative level of other movement behaviours played a role and if the same associations were found in older adults.

## 2. Materials and Methods

### 2.1. Participants

Participants were adults (18–64 years) and older adults (65–79 years) from the first (2007–2009), second (2009–2011) and third (2012–2013) cycles of the CHMS [22]. The CHMS is an ongoing repeated cross-sectional survey that collects various health measures on a nationally representative sample of Canadians living in private households through an interview in participants’ homes and a physical exam in a mobile examination centre. Appointments at the mobile examination centre were either fasting (≥10 h) or non-fasting based on random assignment to allow for fasting blood samples to be collected for the analysis of biochemical measurements requiring a fasted sample [23]. At the end of the appointment, ambulatory participants were fitted with an accelerometer to wear on an elastic belt over their right hip for 7 consecutive days during waking hours. Ethics approval was obtained from Health Canada and the Public Health Agency of Canada Research Ethics Board (reference REB-2005-0025) [24]. All participants provided written informed consent. Further details about the CHMS is available elsewhere [25,26]. A total of 10,217 CHMS participants (8314 adult and 1903 older adult) aged 18–79 years were eligible for this study, however after eliminating records with incomplete data the final analytical sample consisted of 7776 participants (6322 adults and 1454 older adults).

### 2.2. Sedentary Behaviour, Physical Activity and Sleep

Sedentary behaviour, LPA and MVPA were derived from Actical accelerometers (Philips Respironics, Bend, OR, USA). Actical accelerometers have shown higher intra- and inter-instrument reliability than other accelerometer brands (RT3-intra- and interinstrument CV > 40%; Actical − CVintra = 0.5%, CVinter = 5.4%; Actigraph − CVintra = 3.2%, CVinter = 8.6%) [27]. Data were collected in 1-minute epochs. Non-wear time was defined as ≥60 minutes of consecutive minutes of zero counts, with allowance for 1 to 2 minutes of counts between 0 and 10 [28]. Participants were included in the analyses if they had ≥4 valid days, with a valid day being defined as ≥10 h of wear time. Based on validated cut-points in adults, sedentary time (properly stationary time in line with the latest classification [29]) was defined as <100 counts per minute (cpm), LPA as 100–1534 and MVPA as ≥1535 [30,31].

Sleep duration was measured via self-report as part of the in-home interview. Participants were asked, “How many hours do you usually spend sleeping in a 24-h period, excluding time spent resting?” Study interviewers recorded responses to the nearest half hour. Minutes per night of sleep was calculated by multiplying the response by 60.

### 2.3. Health Indicators

Health indicators were selected based on the data availability in the CHMS to represent adiposity, cardiometabolic health, fitness and mental health. The adiposity indicators were body mass index (BMI; full sample) and waist circumference (cycles 2 and 3). Heights and weights were objectively measured following the Canadian Physical Activity, Fitness and Lifestyle Approach (CPAFLA) 3rd edition protocols and BMI (kg/m^2^) was calculated [32]. Waist circumference (cm) at the level of the iliac crest was objectively measured following the National Institute of Health protocols [33]. Cycle 1 waist circumference data was not included because it was measured using different protocols [34].

Fitness indicators were aerobic fitness (cycles 1 and 2) and grip strength (full sample). Aerobic fitness (mL/kg/min) was objectively measured using the modified Canadian Aerobic Fitness Test (mCAFT) following the CPAFLA 3rd edition protocols [32]. Grip strength (kg) was objectively measured using a hand dynamometer following the CPAFLA 3rd edition protocols [32]. Aerobic fitness was not measured in cycle 3.

The cardiometabolic health indicators were systolic blood pressure (full sample), diastolic blood pressure (full sample), resting heart rate (full sample), high-density lipoprotein (HDL)-cholesterol (fasting sub-sample), low-density lipoprotein (LDL)-cholesterol (cycles 2 and 3, fasting sub-samples), C-reactive protein (CRP; fasting sub-sample), triglycerides (fasting sub-sample), insulin (fasting sub-sample), glucose (cycles 2 and 3, fasting sub-samples). Blood pressure (mmHg) and resting heart rate (beats per minute) were objectively measured using an automated oscillometric device following a CHMS-specific protocol that included 6 repeated measurements [35]. HDL-cholesterol (mmol/L), CRP (mg/L) and triglycerides (mmol/L) were measured in serum in all 3 cycles. Insulin (pmol/L) was also measured in serum in all 3 cycles; however, a correction equation was applied to the insulin data in cycle 1 because different methods and instruments were used [36]. Glucose (mmol/L) was measured in serum in cycles 2 and 3. Cycle 1 glucose data was not included because it was measured in plasma and no correction equation was available. LDL-cholesterol (mmol/L) was derived from the Fridewalk equation using serum triglycerides, HDL-cholesterol and total cholesterol in cycles 2 and 3 [37]. Cycle 1 LDL-cholesterol data was not included because it was measured in serum and no correction equation was available.

The mental health indicator was self-reported mental health (full sample). At the in-home interview, participants were asked: “In general, would you say your mental health is …?” There were five response options (poor, fair, good, very good, excellent). For descriptive purposes, fair and poor were combined due to low cell counts for older adults. 

### 2.4. Covariates

Based on previous research on the associations of physical activity, sedentary behaviour and sleep with health in adults and older adults as well as data availability in the CHMS, a number of covariates were included in the analyses to control for confounding effects. Demographic covariates included age (years), sex (male, female) and education (10 categories ranging from “grade 8 or lower” (typically aged 14 or under) to “university degree or certificate above bachelor’s degree” and recoded into 4 categories for descriptive purposes). Health behaviour covariates included smoking status (yes, no) and alcohol consumption (number of drinks per day). Health status covariates included chronic condition (yes, no) and self-rated health (poor, fair, good, very good, excellent). All covariates were measured via self-report as part of the in-home interview.

### 2.5. Statistical Analysis

Data were analysed using the compositional regression approach for physical activity data presented in Chastin et al. [10] using isometric log-ratio (ilr) transformations of the time-use composition as explanatory variables and the health indicators as response variables. This way the association of each movement behaviour with the health indicators is adequately measured in terms relative to the other behaviours (formally through log-ratios) in accordance to the intrinsic co-dependence between the corresponding amounts of time derived from the 24-h constraint. In particular, so-called pivot ilr coordinates were used so that, by means of a sequence of four ilr transformations of the entire time-use composition (leading to three pivot coordinates each from a 4-component time-use composition), the importance of each one of the four movement behaviour components relative to the geometric average of the remaining ones was isolated in turn in the first pivot coordinate to statistically assess its association with the health indicators by regression analysis, for example, the first pivot coordinate linked to MVPA was

(1) $$\sqrt{\frac{3}{4}}\;\ln\frac{\mathrm{MVPA}}{{\left(SB\;.\;LPA\;.\;Sleep\right)}^{1/3}}$$

Four regression models were then fitted to the successive sets of pivot coordinates along with the covariates and the focus was on the coefficient and statistical significance of the first pivot coordinates in each case.

All statistical analyses were performed using SAS version 9.3 (SAS Institute, Cary, NC, USA). Graphical representations were produced using the R statistical system version 3.4.1 (R Foundation for Statistical Computing, Vienna, Austria). Statistical test significance was concluded at the usual 0.05 significance level.

BMI, waist circumference, aerobic fitness, systolic blood pressure, diastolic blood pressure, HDL-cholesterol, LDL-cholesterol, triglycerides, insulin, glucose and CRP were log-transformed to approximate the assumption of normality of the residuals in the regression models. To ensure that the sample was representative of the Canadian population, accelerometer survey weights for combined cycles were used for all analyses. The survey weights accounted for non-response and incomplete accelerometer data. As outlined in the CHMS data user guide, the bootstrap technique [38,39] was used to estimate standard errors and coefficients of variation using degrees of freedom specified by Statistics Canada to account for the survey design (two cycles: 24 degrees of freedom; three cycles: 35 degrees of freedom).

## 3. Results

**Figure 1:**
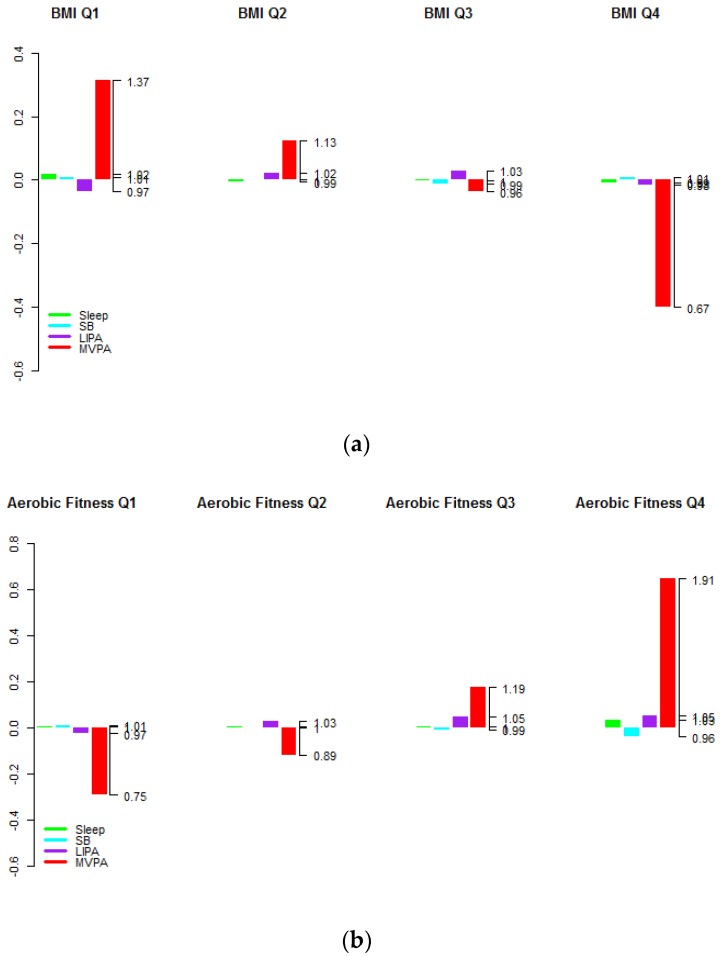

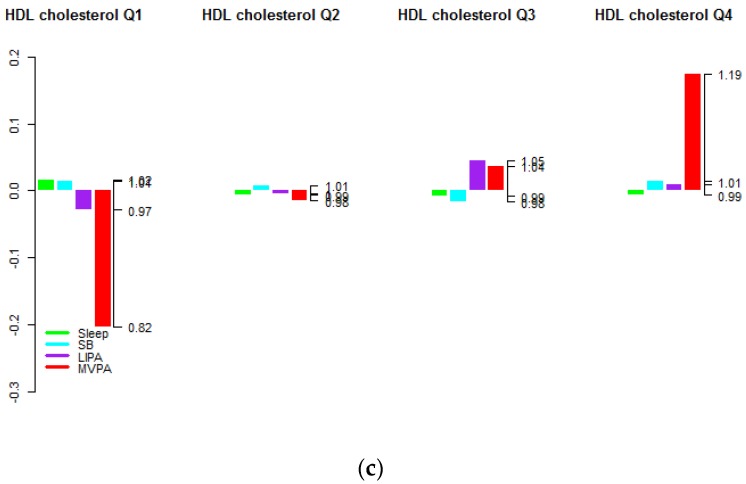
Average time-use compositions of the 24-h day by health indicator quartiles for adults aged (18–64) for (**a**) BMI, (**b**)Aerobic fitness and (**c**) HDL cholesterol level (in log-ratio scale and relative to the overall average time-use composition at the zero baseline). The left axis gives the log-ratio value and the right axis gives the actual proportion relative to the mean composition (e.g., 1.19 means 1.19 times the compositional mean or a proportion higher by 19%).

**Figure 2:**
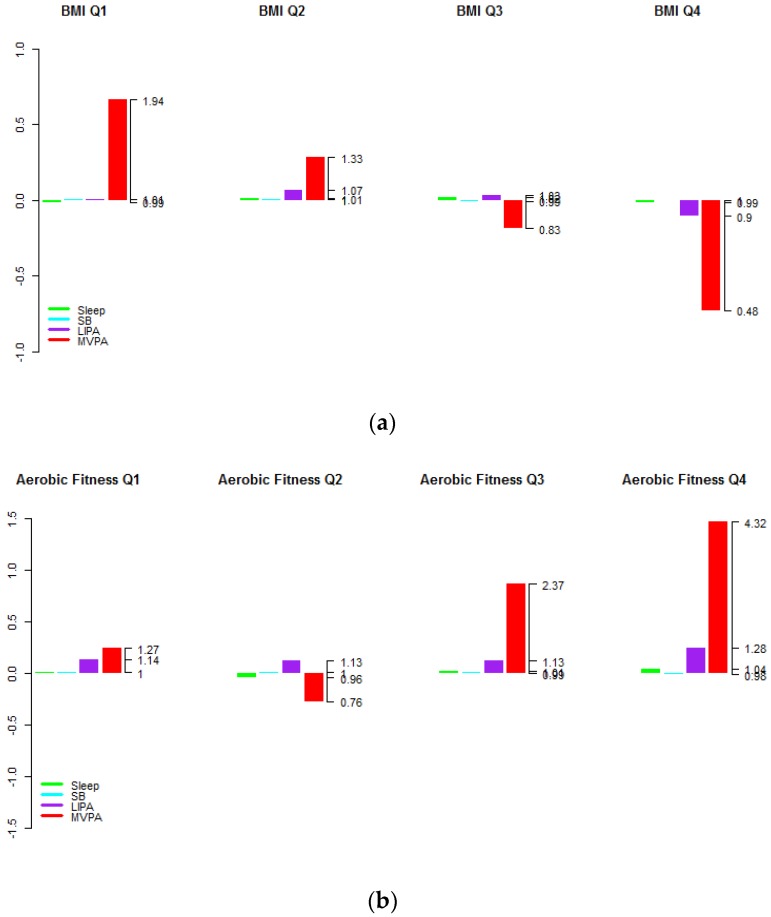

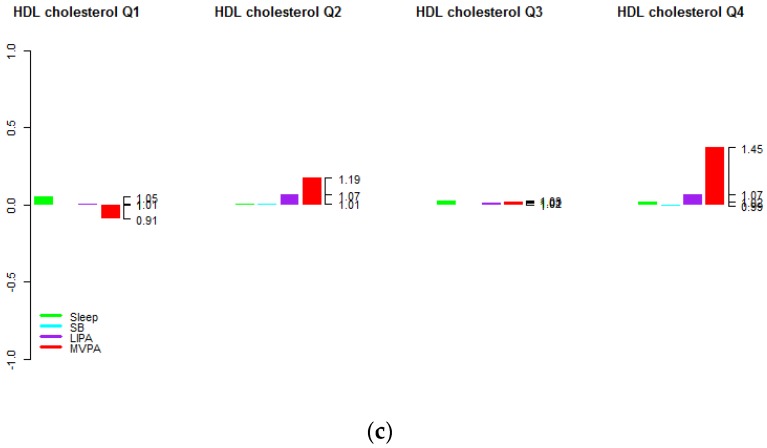
Average time-use compositions of the 24-h day by health indicator quartiles for older adults aged (65–79) for (**a**) BMI, (**b**)Aerobic fitness and (**c**) HDL cholesterol level (in log-ratio scale and relative to the overall average time-use composition at the zero baseline). The left axis gives the log-ratio value and the right axis gives the actual proportion relative to the mean composition (e.g., 1.45 means 1.45 times the compositional mean or a proportion higher by 45%).

A total of 6322 adults (2833 in the fasting sub-sample) and 1454 (697 in the fasting sub-sample) older adults had complete data and were included in the analyses. The sample characteristics are summarized in the supplementary materials. The average time-use compositions of the 24-h day for the quartiles of BMI, aerobic fitness and HDL are shown for adults in Figure 1 and older adults in Figure 2 in log-ratio scale and relative to the overall average time-use composition represented at the zero baseline for reference. The regression coefficients (denoted by the symbol γ) associated with the first pivot coordinates and the *p*-values are presented per health indicator for adults in Table 1 and older adults in Table 2. From an overall view of the results, it is immediately apparent that larger proportions of time in MVPA relative to other components of the time-use composition are almost universally associated with better levels of these health outcomes. Associations with predominance of LPA or sleep relative to other behaviours in the time-use composition are much less straightforward and no statistically significant associations were found with predominance of the SB component relative to other components for both adults and older adults (*p* > 0.05 for all indicators).

In terms of obesity markers, higher proportions of MVPA relative to the other components were associated with lower BMI and waist circumference (*p* < 0.001). The associations between LPA and obesity markers were not clear.

From Figure 1 and Figure 2 it is apparent that predominance of MVPA but also (to a lesser extent) the predominance of LPA relative to other behaviours was positively associated with higher aerobic fitness. However, this latter association was not statistically significant after accounting for other covariates (*p* = 0.121 for adults and *p* = 0.333 for older adults).

An association was found between higher proportions of MVPA relative to other components and higher HDL cholesterol levels in adults only (*p* < 0.001 in adults and *p* = 0.195 in older adults). Figure 1 and Figure 2 also show that individuals in higher quartiles compared to lower quartiles of HDL cholesterol levels had higher proportions of MVPA. Note that analogous graphs for the remaining health indicators can be found in the supplementary materials.

No association was found between the 24-h movement behaviours and blood pressure in adults or older adults (*p* > 0.05 for all first pivot coordinates and both systolic and diastolic blood pressure). The graph of average time-use composition by quartiles (see supplementary materials) shows that individuals in the upper quartiles had higher levels of MVPA relative to other behaviours, however note that this included no adjustment for covariates.

Associations were found between higher proportions of MVPA relative to other behaviours and lower resting heart rate (*p* = 0.012 in adults and *p* = 0.007 in older adults), lower triglycerides level (*p* = 0.004 in adults and *p* = 0.006 in older adults), lower insulin level (*p* < 0.001 in adults and older adults), lower glucose level (*p* = 0.019 in adults and *p* = 0.003 in older adults) and lower CRP level (*p* = 0.005 in adults and *p* < 0.001 in older adults). The graph of average time-use composition by quartiles for blood glucose in the supplementary materials shows that individuals in the lowest quartile had slightly lower levels of MVPA than those in the second lowest quartile. The graphs for resting heart rate, triglycerides level, insulin level and CRP level were broadly consistent with these results.

Associations were also found in adults only between higher proportions of sleep relative to other components and lower HDL cholesterol level (*p* = 0.019), lower LDL cholesterol level (*p* = 0.015), higher insulin level (*p* = 0.016), higher CRP level (*p* < 0.001) and lower grip strength (*p* = 0.003). A marginally statistically significant association was found in older adults between higher proportions of sleep and higher resting heart rate (*p* = 0.041). The graphs of average time-use composition by quartile did not show any clear patterns for any of these outcomes.

Associations were found between higher proportions of LPA relative to other components and lower triglycerides level in adults only (*p* = 0.035), higher BMI in adults only (*p* = 0.002) and higher grip strength in both groups (*p* = 0.019 in adults and *p* = 0.003 in older adults). The graphs of average time-use composition by quartile in the supplementary materials were generally consistent with these results.

Finally, an association was found in older adults between higher proportions of MVPA and better self-rated mental health. The corresponding graph of average time-use composition by quartile (see supplementary materials) was broadly consistent with these results.

Contrasting Table 1 and Table 2, it is evident that many of the associations between the 24-h movement composition and health indicators were weaker in the older adult population. More specifically, many of the associations of relative time allocation to MVPA with health indicators (lower BMI, lower waist circumference, lower resting heart rate and lower blood glucose) were similar between adults and older adults, whereas other associations (CRP, insulin, triglycerides) were weaker, although they retain broadly similar levels of statistical significance due to lower variation in outcome among the older population. The associations with HDL cholesterol and aerobic fitness declined to a level that was no longer statistically significant.

## 4. Discussion

The results reported broadly agree with previous findings on the associations between physical activity and health indicators in adults and older adults. The beneficial association between MVPA and obesity markers [40] and aerobic fitness [41], in particular, are well-established. However, it is possible to do a lot of MVPA and still engage in several hours of daily sedentary behaviour. Recently it was questioned whether the beneficial association with MVPA is attenuated by too much time spent sedentary or sleep or in light activity. This study shows that this is not the case but that it is the time spent in MVPA relative to the other behaviour that is associated with better health outcome rather than the absolute amount of time spent in MVPA. Other contrasting results are worth noting.

One notable contrasting result is with blood pressure where no association was observed. This is the third compositional analysis in adults, to our knowledge, where no association was found between MVPA (relative to other behaviours) and blood pressure. In the US NHANES 2005-06 data [9], the only significant association found was a negative association with time allocated to sleep relative to other behaviours and in more recent work on the Health Survey for England 2008 data (waking day only) no significant association was found [42]. Conventional analytical approaches generally support the existence of such an association [43,44,45], although the evidence for a dose-response relationship is more mixed [46]. At present, the compositional evidence does not support the association with blood pressure and given these contrasting findings (as opposed to adiposity where compositional models tend to be broadly in line with existing analytical approaches) there could be significant value in future research in compositional analysis in movement behaviours exploring systolic and diastolic blood pressure. We note that blood pressure measurements in CHMS are taken using automated devices [35] to reduce “white coat hypertension”—the elevated blood pressure associated with the presence of the health care professional and the procedures of measurement [47], which may affect comparisons with other studies.

Our results attribute a stronger association between MVPA and the levels of triglycerides, blood glucose and blood insulin than comparable results based on NHANES [10]. Both studies attributed beneficial associations to MVPA but our findings are statistically significant, in contrast to the NHANES results. These differences might be attributable to differences in the populations of the two countries, or the use of different monitors by the NHANES study (Actigraph 7164; Actigraph, LLC, Pensacola, FL, USA).

The findings for 24-h movement behaviours other than MVPA are mixed. In particular, the allocation to SB relative to other behaviours showed no statistically significant associations, in contrast to previously mentioned compositional studies, particularly with respect to adiposity indicators. Nevertheless, our results demonstrate that some of the indicators showed clear associations with relative allocation of time to other behaviour types (sleep and LPA), indicating the importance of considering the composition of the whole day, rather than isolated behaviours. Of particular note was the strong negative association between sleep, relative to other behaviours and cholesterol levels. The importance of considering the behaviour type(s) replaced, not just the behaviour type increased, for example, the associations of LPA will depend on whether they replace SB or MVPA, is now widely accepted and compositional analyses account for this, in addition to their other benefits (e.g., sub-compositional coherence and scale invariance) [48].

An interesting feature of the results is that the response to physical activity seems to be blunted in later life. This observation is well documented in the exercise physiology literature and while raising the levels of exercise might offset the effects of secondary aging, the persistency of these benefits is less certain [49,50]. This feature is particularly notable for aerobic fitness and HDL cholesterol levels. The association was also diminished for triglycerides, insulin, glucose and CRP, however these are commensurate with the lower variation observed among older adults. Figure 1 illustrates that the differences may be more attributable to differences in the physical activity patterns of less healthy individuals, however this is speculative as this graph is not adjusted for the other covariates.

Our findings are based on a large nationally representative sample; however, this is a cross sectional study and there might be a variety of possible explanations for the differences in response between adults and older adults. The difference could potentially be the effect of aging (due to muscular atrophy [49]) but it could also be attributable to other differences between the two cohorts, such as diet or socioeconomic factors not accounted for in the study. Alternatively, there may be differences in the quality of MVPA between the two age groups that are not captured in our data (such as the length of bouts of physical activity [51], or the extent to which the activity boosts endurance and/or resistance [49]). This is the first study, to our knowledge, to apply compositional techniques separately to adults and older adults in the same sample. Experimental evidence would be needed to confirm any such effect but if confirmed it suggests that interventions would potentially need to target different behaviour allocations in older adults.

Finally, we note that higher proportions of MVPA appear to be associated with better mental health in older adults (whereas the association is quite weak among adults aged 18–64). The benefits of physical activity to mental health can be controversial, however most studies support improvements in self-assessed mental health, albeit the findings for more rigorous batteries of psychological tests are often not significant [52]. Again, we stress the cross-sectional nature of the study and that a variety of explanations might be behind the association. At a high level, it may be that physical activity improves mental health in older adults, which suggests a source of decline commencing at older ages that can be arrested by higher levels of physical activity. It may also be that better mental health encourages physical activity [53], or there may be some mediating variables linking the two. For example, social isolation has been shown to have a negative association with both self-assessed mental health [54] and physical activity [55] in older adults.

### Strengths and Limitations

The strength of this analysis is applying a compositional approach applied to a large nationally representative dataset, in which most movement behaviours are measured objectively. The compositional approach ensures that estimates are fully adjusted for all time use and allow us to explore the combined and synergistic associations of the different behaviours. The main limitation of the analysis is that the underlying study is cross-sectional, therefore any causal inference is limited and estimated associations may reflect a population shift in time-use composition rather than actual effects for individuals. In the case of adiposity outcomes reverse causality is known to play a role and so associations might also be inflated. This type of compositional analysis should be repeated on longitudinal data to provide evidence of individual change, more accurate estimates and demonstrate potential causality. Inevitably there are limitations in respect of the measurement of physical activity. Firstly, similar to all hip-worn monitors, the Actical monitor used in this study cannot discriminate between postural sitting and standing [56,57]. Therefore, it is possible that the proportion of time allocated to SB and LIPA are not accurate and that SB time has been over estimated. Secondly, time allocated to sleep has been assessed from self-reported data, which is likely to be less accurate than objective data obtained from the Actical monitor. Lastly, we note that the accelerometer thresholds used in our analysis to define the different behaviour types were fixed values and were not adjusted for the different age groups. In particular, the proportion of time allocated to LIPA and MVPA in older adults may not be accurate.

## 5. Conclusions

In this nationally representative sample with objectively-measured daily pattern of activity we found evidence of the importance to health of the distribution of time across the movement behaviour composition of the whole day. Higher daily time allocations to MVPA relative to other movement behaviours tended to be associated with better health indicators, however other movement behaviours also play a role. While it has long been known that time spent in MVPA and to some extend in LIPA is beneficial for health, recent research questioned whether this remained when time spent in other movement behaviour is considered. This study confirms that MVPA remains associated with better health outcomes when the full 24 movement behaviour spectrum is taken into account. In addition, the association was shown to be weaker in older adults. This hints that the response to physical activity might be blunted with age.

## Figures and Tables

**Table 1:** Associations between health indicators and reallocations of time to individual components of the 24-h day from across the other components of the 24-h day for adults (18–64 years).

Health Indicator	SB	*p*-Value **	LPA	*p*-Value **	MVPA	*p*-Value **	Sleep	*p*-Value **
BMI *	0.001	0.984	**0.052**	**0.002**	**–0.033**	**<0.001**	–0.020	0.599
Waist circumference *	0.007	0.838	0.031	0.051	**–0.025**	**<0.001**	–0.012	0.736
Aerobic fitness	−3.100	0.664	8.295	0.121	**11.572**	**<0.001**	**–16.767**	**0.034**
Systolic blood pressure *	0.010	0.373	0.010	0.231	–0.005	0.134	–0.015	0.357
Diastolic blood pressure *	0.007	0.514	0.018	0.066	0.002	0.711	–0.027	0.117
Resting Heart Rate	1.128	0.301	–0.136	0.865	**–0.764**	**0.012**	–0.227	0.884
HDL Cholesterol Level *	–0.032	0.447	0.018	0.604	**0.042**	**<0.001**	**–0.092**	**0.019**
LDL Cholesterol Level *	0.089	0.148	0.062	0.090	0.006	0.695	**–0.157**	**0.015**
Triglycerides Level *	0.074	0.438	**–0.119**	**0.035**	**–0.059**	**0.004**	0.105	0.327
Insulin Level *	–0.063	0.440	–0.064	0.349	**–0.116**	**<0.001**	**0.243**	**0.016**
Glucose Level *	–0.035	0.106	0.003	0.849	**–0.021**	**0.019**	0.053	0.078
C-reactive Proteins Level *	–0.150	0.509	–0.156	0.164	**–0.162**	**0.005**	**0.687**	**<0.001**
Grip Strength	0.626	0.726	**3.183**	**0.003**	–0.370	0.356	**–0.497**	**0.003**
Self-assessed mental health	0.070	0.503	–0.006	0.924	–0.002	0.935	–0.062	0.598

* Log-transformed response variable; ** Ordinary Wald test on first pivot coordinate regression coefficient for each time-use component (SB, LPA, MVPA, Sleep; H_0_: γ = 0); Statistically significant results (*p*-value < 0.05) are shown in **bold**.

**Table 2:** Associations between health indicators and reallocations of time to individual components of the 24-h day from across the other components of the 24-h day for older adults (65–79 years).

Health Indicator	SB	*p*-Value **	LPA	*p*-Value **	MVPA	*p*-Value **	Sleep	*p*-Value **
BMI *	–0.016	0.563	0.002	0.895	**–0.032**	**<0.001**	0.046	0.086
Waist circumference *	–0.008	0.805	–0.006	0.725	**–0.030**	**<0.001**	0.044	0.132
Aerobic fitness	–20.075	0.368	17.057	0.333	6.922	0.083	–3.904	0.805
Systolic blood pressure *	0.003	0.906	0.012	0.325	–0.005	0.137	–0.010	0.709
Diastolic blood pressure *	–0.024	0.366	0.017	0.152	–0.001	0.767	0.008	0.771
Resting Heart Rate	0.067	0.075	0.275	0.781	**–0.769**	**0.003**	**4.185**	**0.041**
HDL Cholesterol Level *	–0.047	0.928	0.021	0.522	0.009	0.195	–0.024	0.719
LDL Cholesterol Level *	–0.150	0.702	–0.013	0.867	0.012	0.646	–0.059	0.159
Triglycerides Level *	1.126	0.506	–0.092	0.167	**–0.038**	**0.006**	0.189	0.069
Insulin Level *	–0.016	0.686	–0.063	0.574	**–0.082**	**<0.001**	0.077	0.685
Glucose Level *	–0.008	0.381	0.012	0.636	**–0.024**	**0.003**	0.059	0.387
C-reactive Proteins Level *	–20.075	0.509	–0.074	0.625	**–0.134**	**<0.001**	0.358	0.109
Grip Strength	0.003	0.677	**2.979**	**0.028**	–0.599	0.213	–3.505	0.182
Self-assessed mental health	–0.122	0.362	0.005	0.952	**–0.042**	**0.037**	0.158	0.298

* Log-transformed response variable; ** Ordinary Wald test on first pivot coordinate regression coefficient for each time-use component (SB, LPA, MVPA, Sleep; H_0_: γ = 0); Statistically significant results (*p*-value < 0.05) are shown in **bold**.

## Supplementary Materials

The following are available online at http://www.mdpi.com/1660-4601/15/8/1779/s1, Table S1: Weighted participant characteristics of the 2007/09, 2009/11, 2012/13 CHMS; Figures S1–S28: Average time-use compositions of the 24 h day by indicator quartiles in log-ratio scale and relative to the overall average time-use composition at the zero baseline.
