# Supplementary material for: Compositional Analysis of the Associations between 24-h Movement Behaviours and Health Indicators among Adults and Older Adults from the Canadian Health Measure Survey
Source: Int J Environ Res Public Health. 2018 Aug 18;15(8):1779. doi: 10.3390/ijerph15081779 (PMC6121426; doi:10.3390/ijerph15081779)
Supplement: Supplementary file 1 [file ijerph-15-01779-s001.pdf]

International Journal of
Environmental Research and Public Health

MDPI

Supplementary Materials: Compositional Analysis of the Associations between 24-Hour Movement Behaviours and Health Indicators among Adults and Older Adults from the Canadian Health Measure Survey

Table S1. Weighted participant characteristics of the 2007/09, 2009/11, 2012/13 CHMS

Variables	Adults (18–64)		Older Adults (65–80)	
	**Full sample** (*n* = 6322)	**Fasting sub-sample** (*n* = 2833)	**Full sample** (*n* = 1454)	**Fasting sub-sample** (*n* = 697)
Age (years)	41.3 (0.2)	41.8 (0.8)	69.3 (0.3)	68.6 (0.4)
Sex (%)				
- Male	50.4	50.0	47.6	50.7
- Female	49.6	50.0	52.4	49.3
Education (%)				
- Less than secondary school graduation	3.6	2.6 [E]	19.0	15.8
- Secondary school graduation	11.3	11.8	16.4	17.5
- Some post-secondary	4.2	3.7	2.7 [E]	3.2
- Post-secondary graduation	80.9	81.9	61.9	63.5
Smoking Status (%)				
- Yes	21.3	21.0	12.2	12.7
- No	78.7	79.0	87.8	87.3
Alcohol Consumption (# of drinks per day)	0 (0.03)	0 (0.05)	0 (0.05)	0 (0.05)
Chronic Condition (%)				
- Yes	59.1	57.8	90.4	91.4
- No	40.9	42.2	9.6	8.6
Self-rated health (%)				
- Poor	2.0 [E]	1.5 [E]	2.7 [E]	1.9
- Fair	7.0	6.1	11.1	8.9
- Good	34.7	34.7	38.7	37.1
- Very Good	40.5	42.4	34.0	39.9
- Excellent	15.8	15.3	13.5	12.2
**Health Indicators**				
BMI (m/kg^2)	26.2 (0.2)	-	27.3 (0.2)	-
Waist Circumference (cm; n=4117 and n=951)‡	91.4 (0.7)	-	97.1 (0.7)	-
Systolic Blood Pressure (mmHg)	108.5 (0.4)	-	123.2 (0.8)	-
Diastolic Blood Pressure (mmHg)	70.4 (0.3)	-	70.8 (0.4)	-
Resting heart rate (beats per minute)	66.8 (0.3)	-	64.6 (0.5)	-
Grip Strength (Age: 18-79; kg; n=4437 and n=937)	68.3 (1.3)	-	55.9 (1.3)	-
Aerobic Fitness (Age: 18-79 years; Fitness score; n=3517 and n=200)†	368.9 (2.9)	-	228.5 (4.8)	-
Self-reported Mental Health (%)		-		-
- Poor/ Fair	5.4	-	3.0	-
- Good	19.8	-	24.9	-
- Very Good	39.8	-	37.3	-
- Excellent	35.0	-	34.8	-
HDL Cholesterol (mmol/L)	-	1.29 (0.02)	-	1.37 (0.03)
LDL Cholesterol (mmol/L; n=2084 and n=485) ‡	-	2.70 (0.05)	-	2.67 (0.08)

C-reactive Protein (mg/L)	-	1.22 (0.07)	-	1.61 (0.12)
Triglycerides (mmol/L)	-	1.11 (0.03)	-	1.25 (0.05)
Insulin (pmol/L)	-	54.78 (1.67)	-	63.74 (2.45)
Glucose (mmol/L; n=3199 and n=726) ‡	-	4.81 (0.03)	-	5.19 (0.05)

HDL = High-density lipoprotein cholesterol; LDL = Low-density lipoprotein cholesterol; Data presented as median (standard error) for continuous variations and percentage for categorical variables; ‡ Waist circumference, LDL Cholesterol, and Glucose includes participants from cycle 2 and 3 only. † Aerobic Fitness included participants from cycle 1 and 2 only. ^{E} = interpret with caution (coefficient of variation 16.6% to 33.3%)

Color Key

Continuous variable presented as median (standard error)
Value of categorical variable presented as percentage

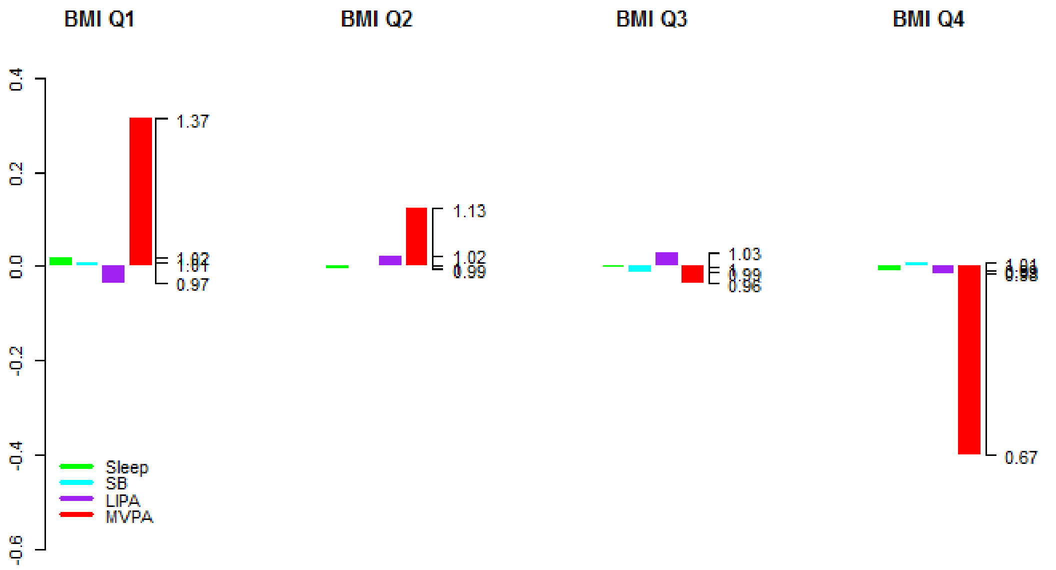

Figure S1. Average time-use compositions of the 24 hour day by BMI quartiles for adults aged (18–64) in log-ratio scale and relative to the overall average time-use composition at the zero baseline. The left axis gives the log-ratio value and the right axis gives the actual proportion relative to the mean composition (e.g. 1.20 means 1.20 times).

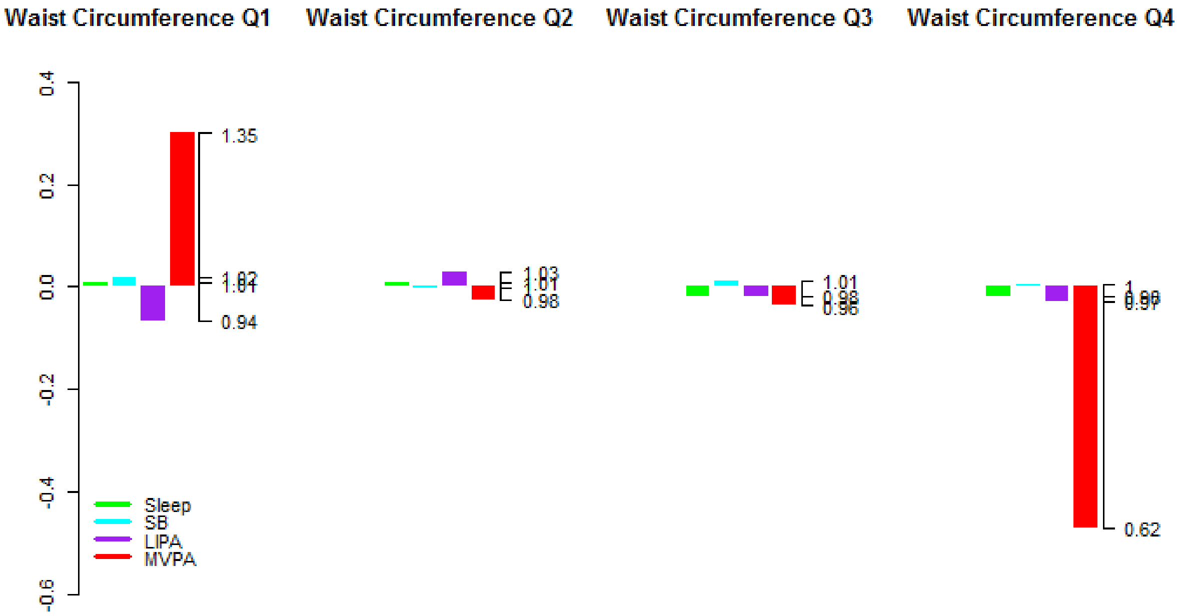

Figure S2. Average time-use compositions of the 24 hour day by waist ircumference quartiles for adults aged (18–64) in log-ratio scale and relative to the overall average time-use composition at the zero baseline. The left axis gives the log-ratio value and the right axis gives the actual proportion relative to the mean composition (e.g. 1.20 means 1.20 times).

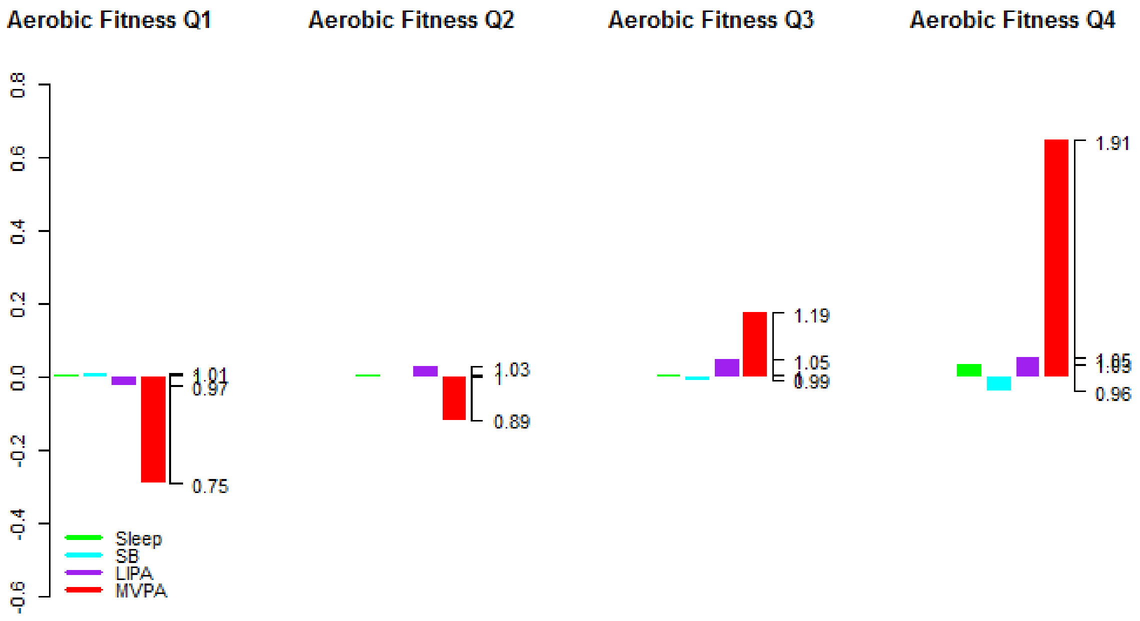

Figure S3. Average time-use compositions of the 24 hour day by VO2 maximum quartiles for adults aged (18–64) in log-ratio scale and relative to the overall average time-use composition at the zero baseline. The left axis gives the log-ratio value and the right axis gives the actual proportion relative to the mean composition (e.g. 1.20 means 1.20 times).

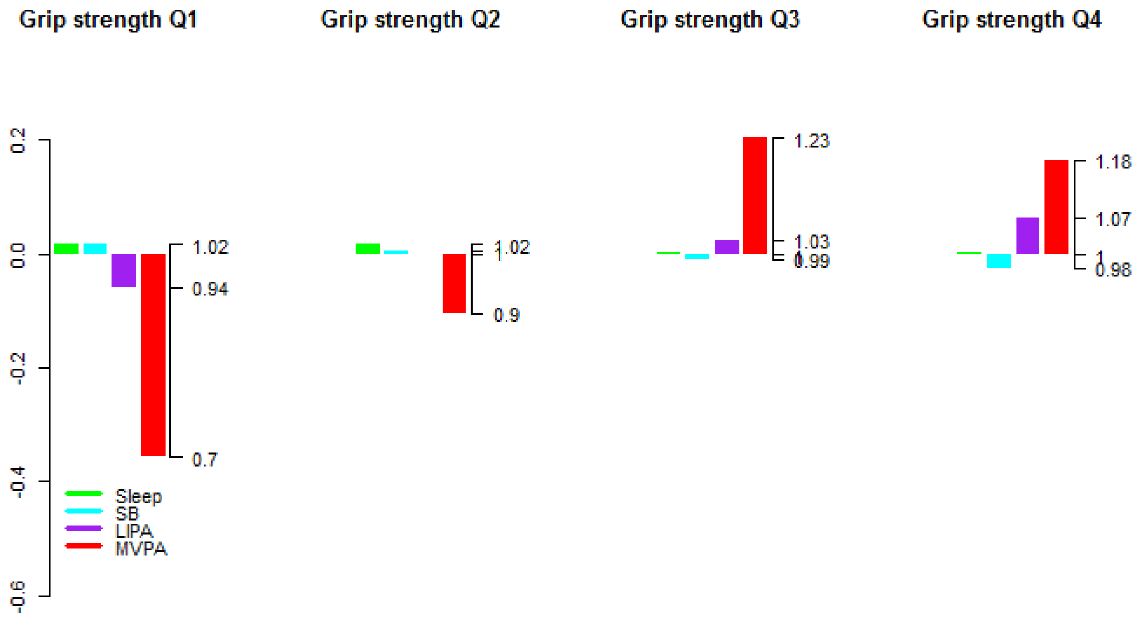

Figure S4. Average time-use compositions of the 24 hour day by grip strength quartiles for adults aged (18–64) in log-ratio scale and relative to the overall average time-use composition at the zero baseline. The left axis gives the log-ratio value and the right axis gives the actual proportion relative to the mean composition (e.g. 1.20 means 1.20 times).

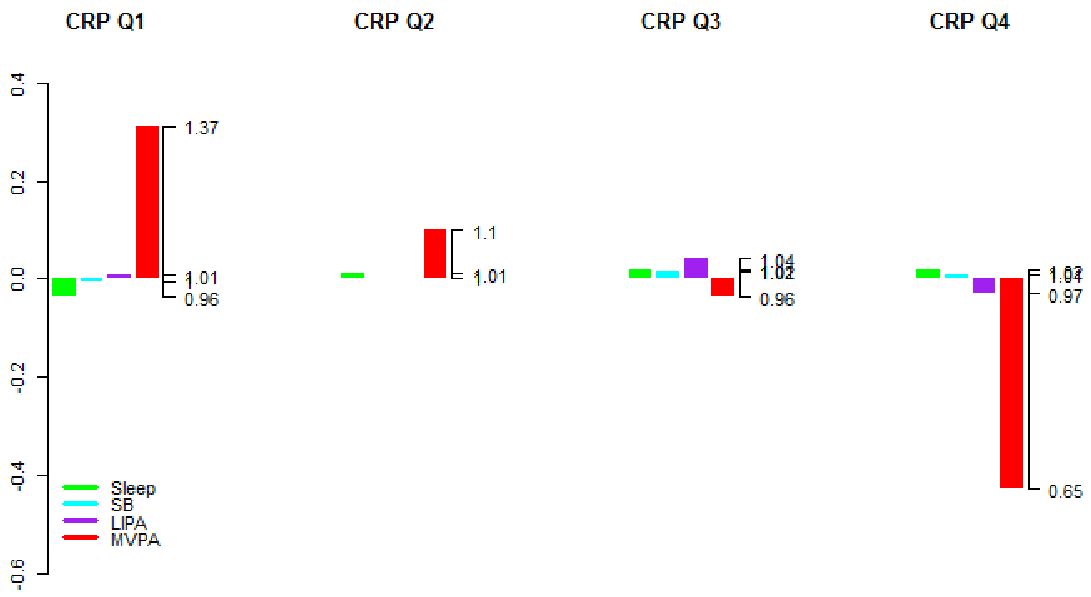

Figure S5. Average time-use compositions of the 24 hour day by CRP quartiles for adults aged (18–64) in log-ratio scale and relative to the overall average time-use composition at the zero baseline. The left axis gives the log-ratio value and the right axis gives the actual proportion relative to the mean composition (e.g. 1.20 means 1.20 times).

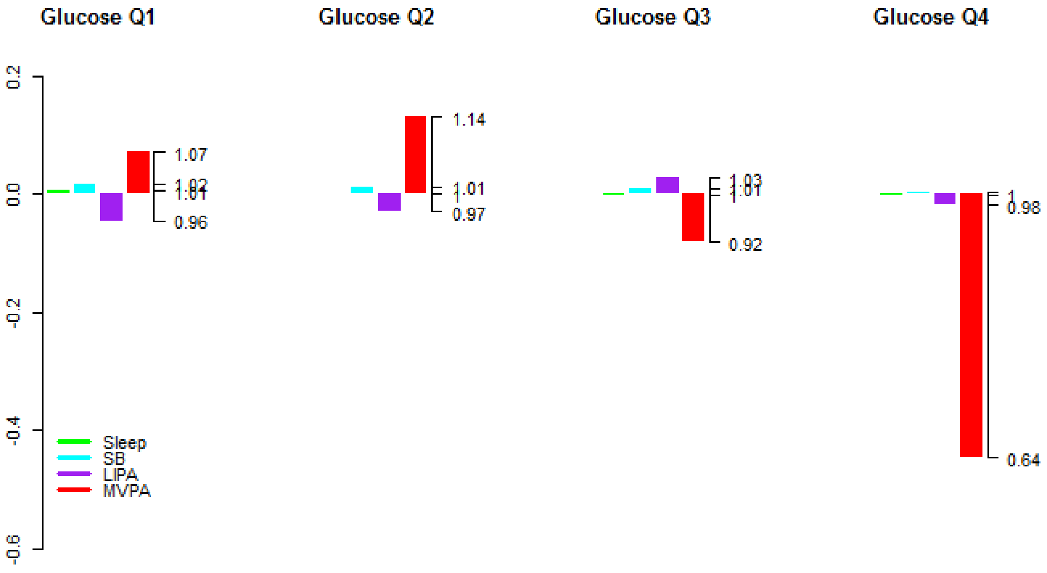

Figure S6. Average time-use compositions of the 24 hour day by blood glucose quartiles for adults aged (18–64) in log-ratio scale and relative to the overall average time-use composition at the zero baseline. The left axis gives the log-ratio value and the right axis gives the actual proportion relative to the mean composition (e.g. 1.20 means 1.20 times).

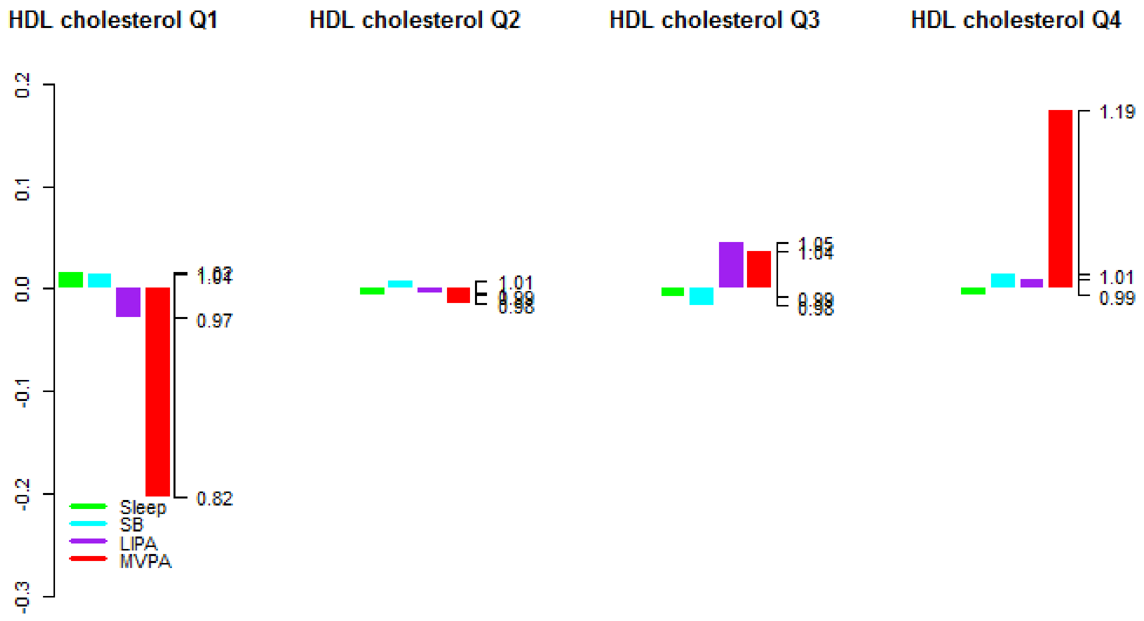

Figure S7. Average time-use compositions of the 24 hour day by HDL cholesterol quartiles for adults aged (18–64) in log-ratio scale and relative to the overall average time-use composition at the zero baseline. The left axis gives the log-ratio value and the right axis gives the actual proportion relative to the mean composition (e.g. 1.20 means 1.20 times).

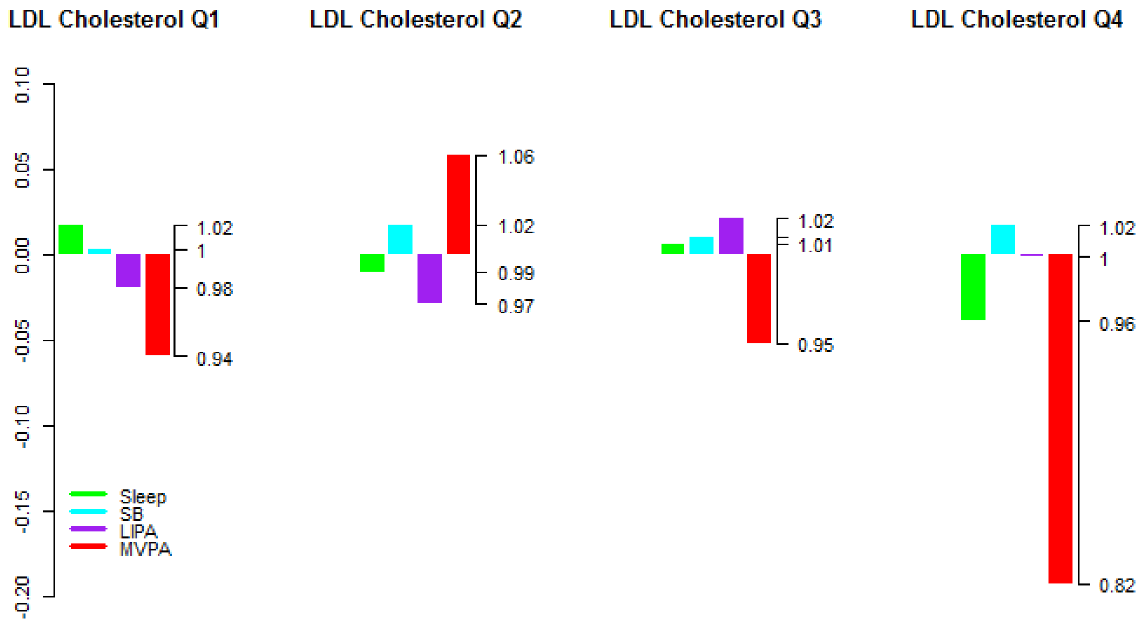

Figure S8. Average time-use compositions of the 24 hour day by LDL cholesterol quartiles for adults aged (18–64) in log-ratio scale and relative to the overall average time-use composition at the zero baseline. The left axis gives the log-ratio value and the right axis gives the actual proportion relative to the mean composition (e.g. 1.20 means 1.20 times).

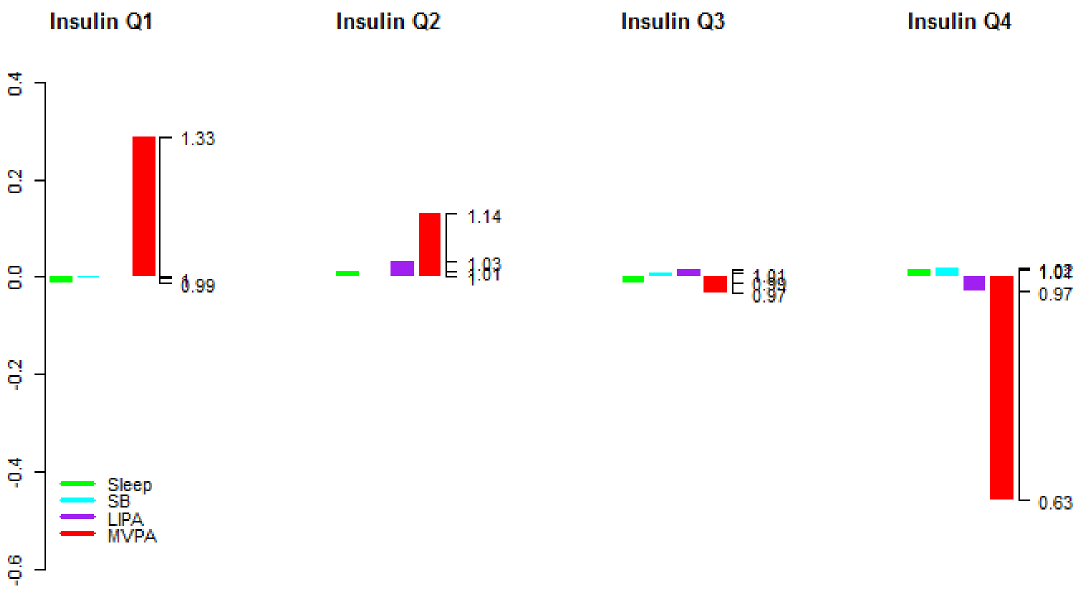

Figure S9. Average time-use compositions of the 24 hour day by insulin quartiles for adults aged (18–64) in log-ratio scale and relative to the overall average time-use composition at the zero baseline. The left axis gives the log-ratio value and the right axis gives the actual proportion relative to the mean composition (e.g. 1.20 means 1.20 times).

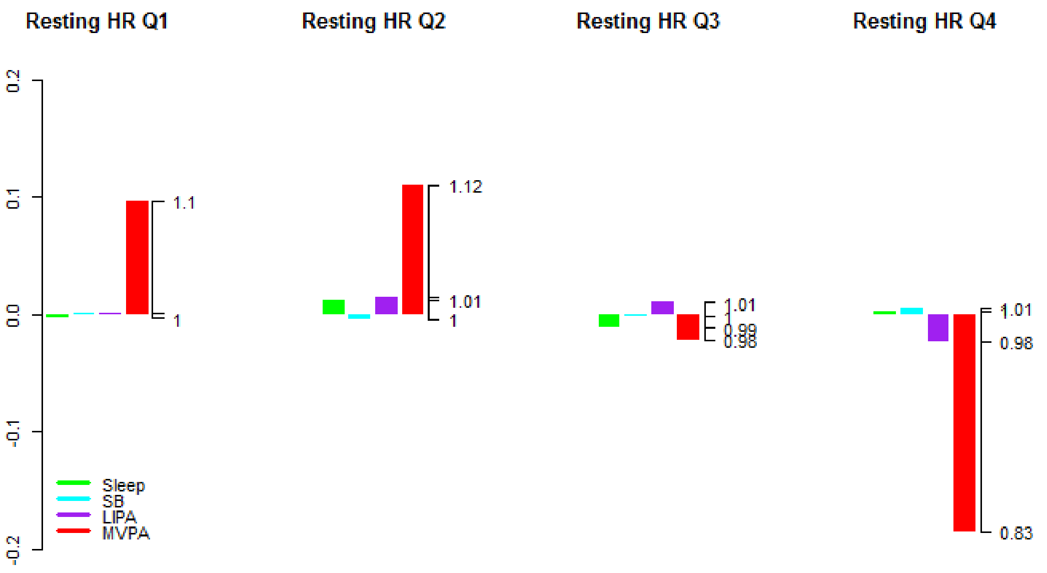

Figure S10. Average time-use compositions of the 24 hour day by resting heart rate quartiles for adults aged (18–64) in log-ratio scale and relative to the overall average time-use composition at the zero baseline. The left axis gives the log-ratio value and the right axis gives the actual proportion relative to the mean composition (e.g. 1.20 means 1.20 times).

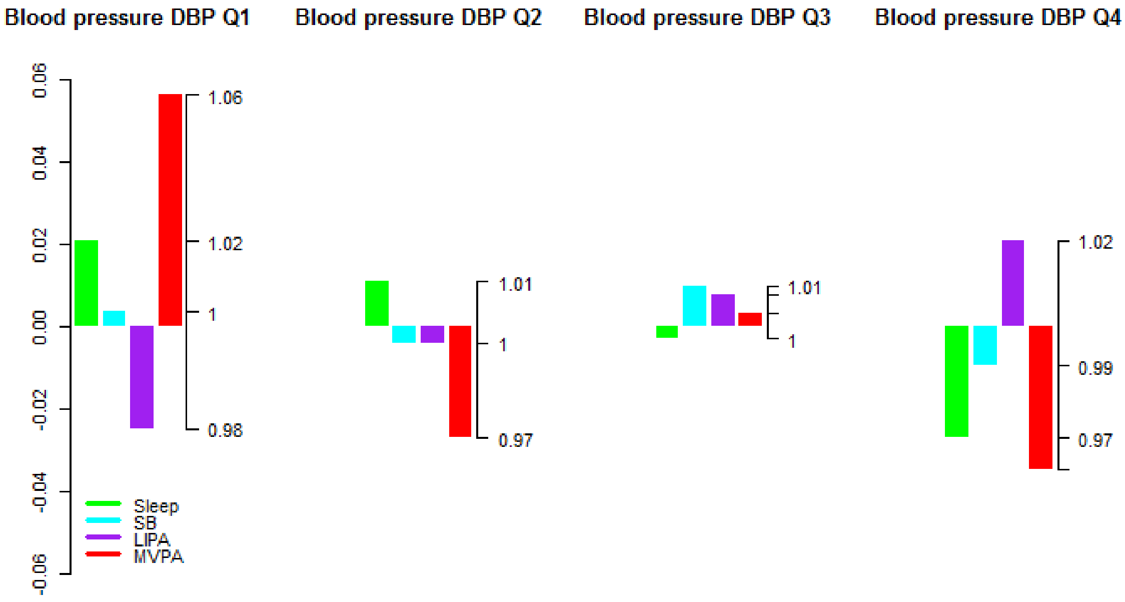

Figure S11. Average time-use compositions of the 24 hour day by diastolic blood pressure quartiles for adults aged (18–64) in log-ratio scale and relative to the overall average time-use composition at the zero baseline. The left axis gives the log-ratio value and the right axis gives the actual proportion relative to the mean composition (e.g. 1.20 means 1.20 times).

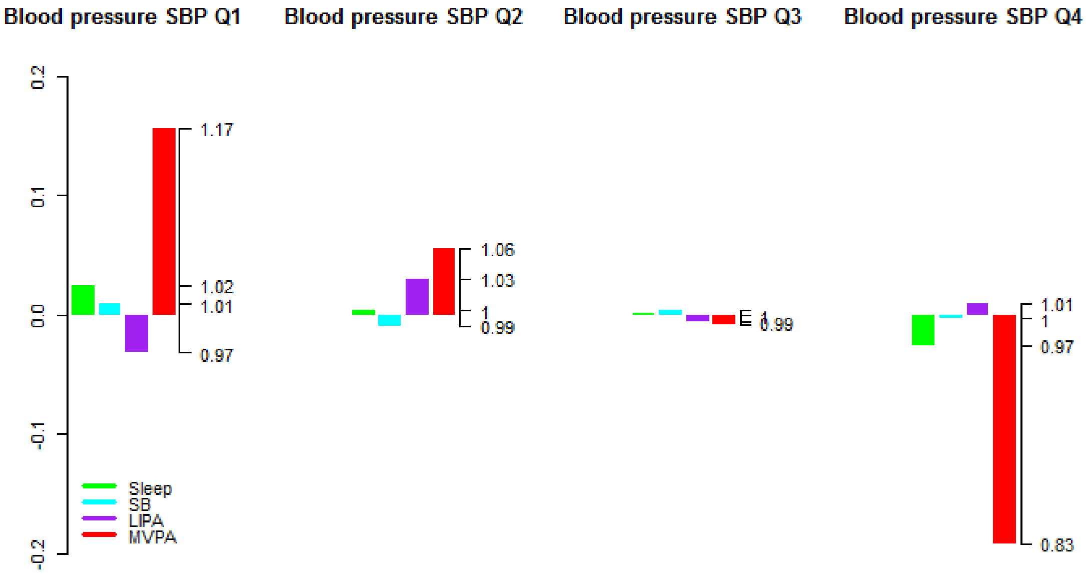

Figure S12. Average time-use compositions of the 24 hour day by systolic blood pressure quartiles for adults aged (18–64) in log-ratio scale and relative to the overall average time-use composition at the zero baseline. The left axis gives the log-ratio value and the right axis gives the actual proportion relative to the mean composition (e.g. 1.20 means 1.20 times).

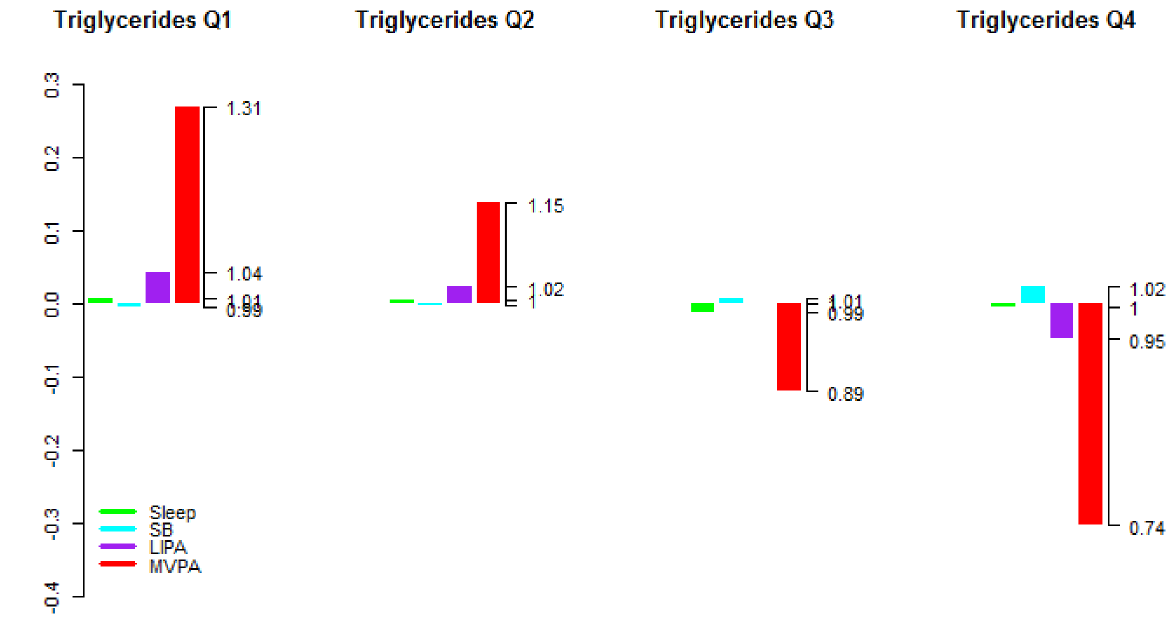

Figure S13. Average time-use compositions of the 24 hour day by triglycerides quartiles for adults aged (18–64) in log-ratio scale and relative to the overall average time-use composition at the zero baseline. The left axis gives the log-ratio value and the right axis gives the actual proportion relative to the mean composition (e.g. 1.20 means 1.20 times).

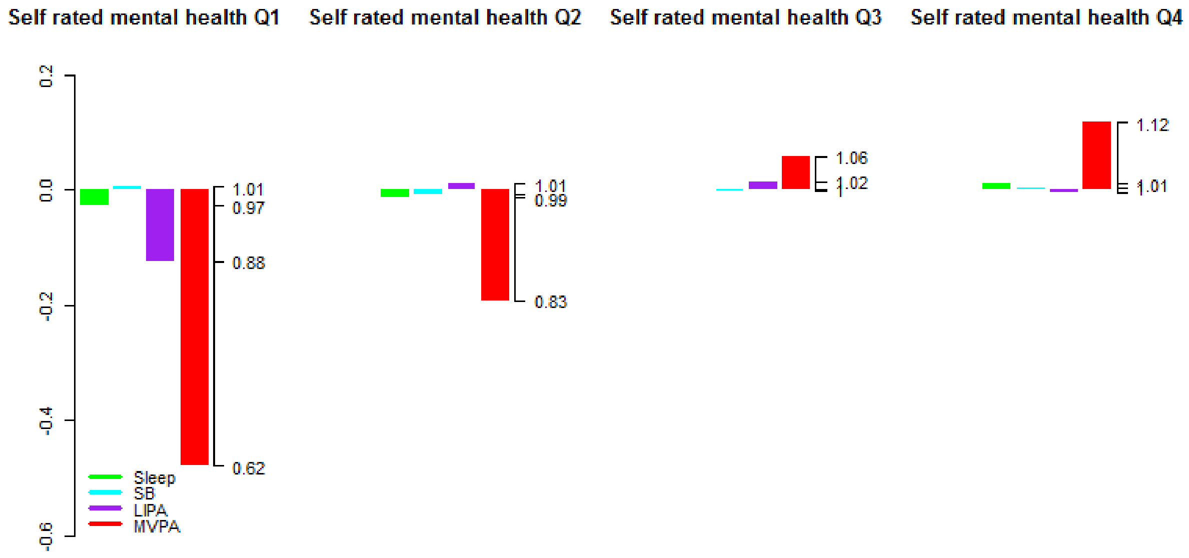

Figure S14. Average time-use compositions of the 24 hour day by self-rated mental health quartiles for adults aged (18–64) in log-ratio scale and relative to the overall average time-use composition at the zero baseline. The left axis gives the log-ratio value and the right axis gives the actual proportion relative to the mean composition (e.g. 1.20 means 1.20 times).

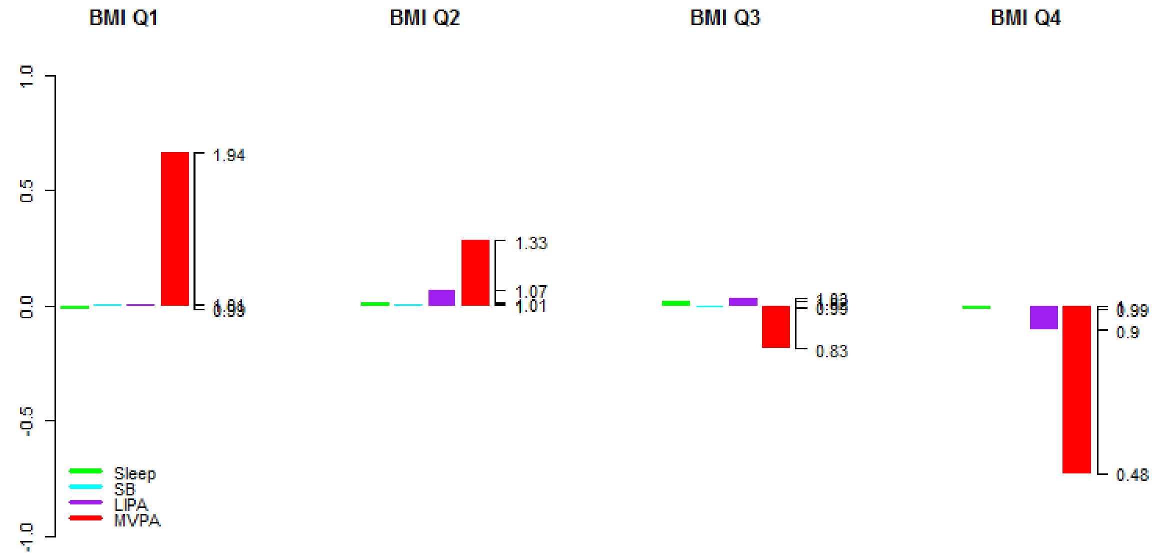

Figure S15. Average time-use compositions of the 24 hour day by BMI quartiles for older adults aged (65-79) in log-ratio scale and relative to the overall average time-use composition at the zero baseline. The left axis gives the log-ratio value and the right axis gives the actual proportion relative to the mean composition (e.g. 1.20 means 1.20 times).

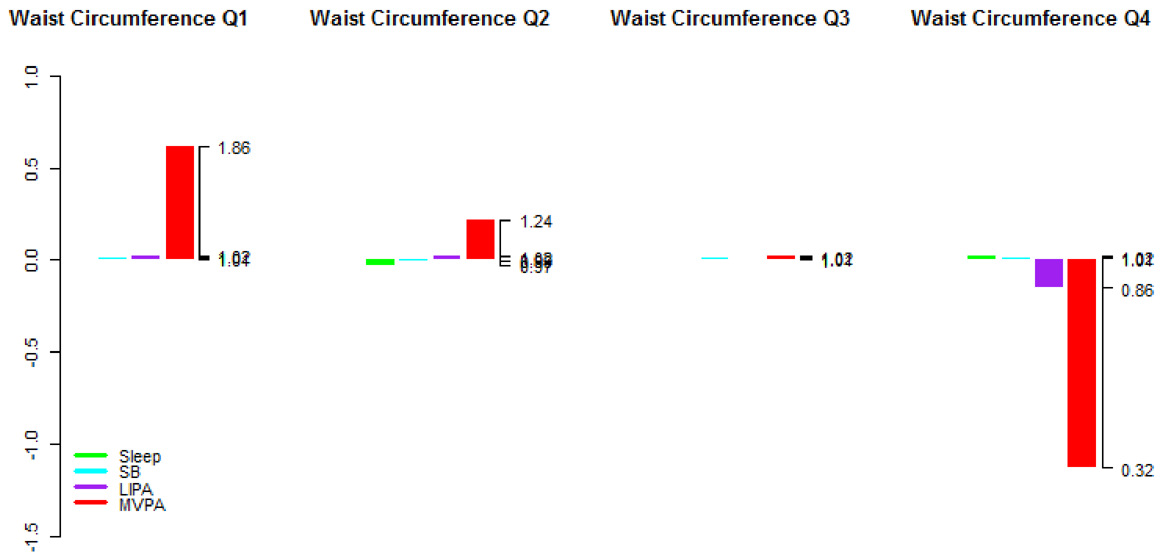

Figure S16. Average time-use compositions of the 24 hour day by waist ircumference quartiles for older adults aged (65-79) in log-ratio scale and relative to the overall average time-use composition at the zero baseline. The left axis gives the log-ratio value and the right axis gives the actual proportion relative to the mean composition (e.g. 1.20 means 1.20 times).

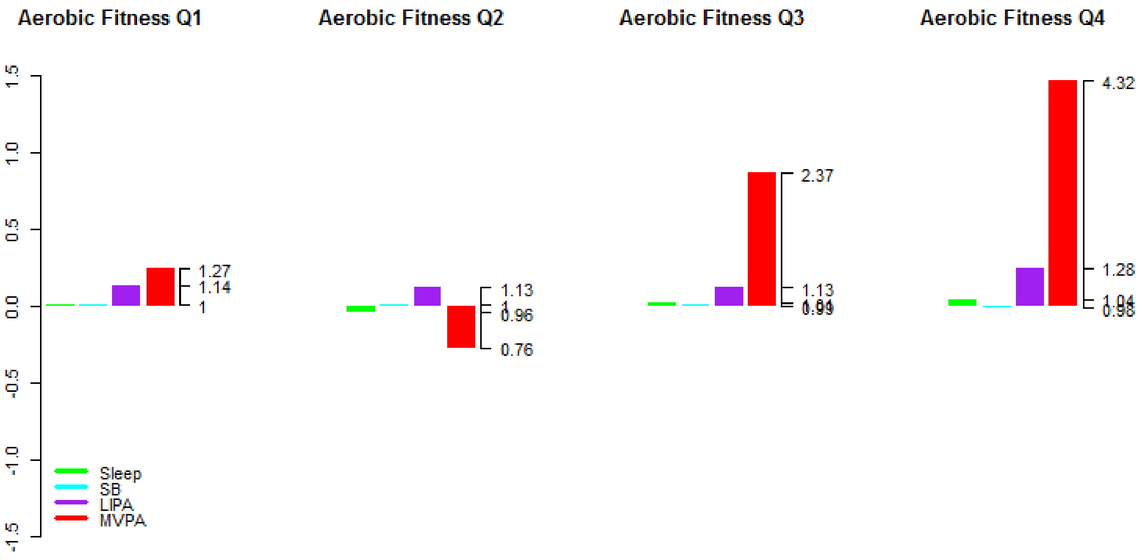

Figure S17. Average time-use compositions of the 24 hour day by VO2 maximum quartiles for older adults aged (65-79) in log-ratio scale and relative to the overall average time-use composition at the zero baseline. The left axis gives the log-ratio value and the right axis gives the actual proportion relative to the mean composition (e.g. 1.20 means 1.20 times).

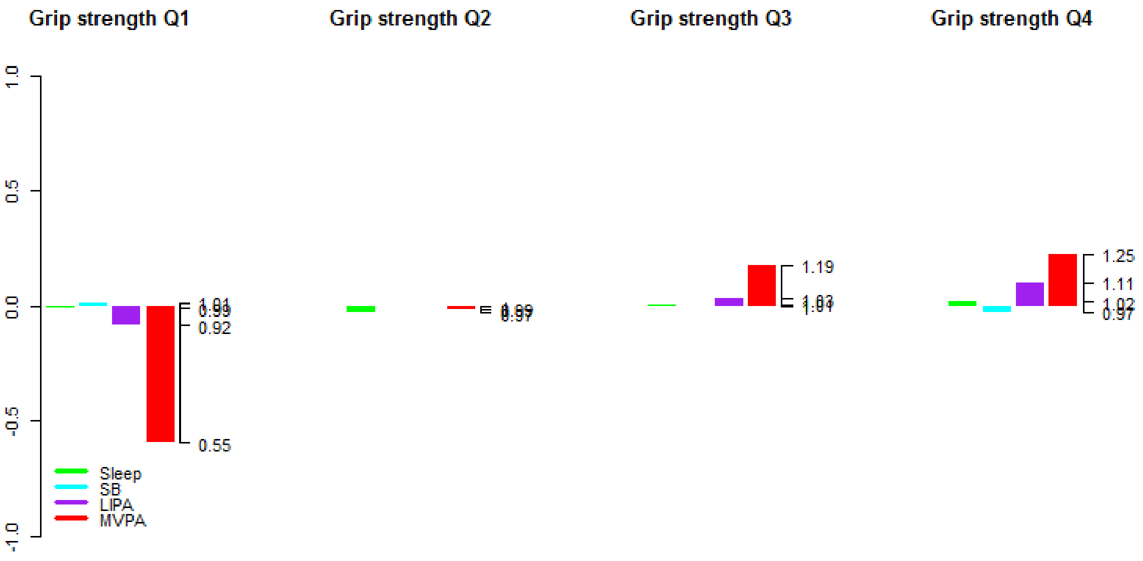

Figure S18. Average time-use compositions of the 24 hour day by grip strength quartiles for older adults aged (65-79) in log-ratio scale and relative to the overall average time-use composition at the zero baseline. The left axis gives the log-ratio value and the right axis gives the actual proportion relative to the mean composition (e.g. 1.20 means 1.20 times).

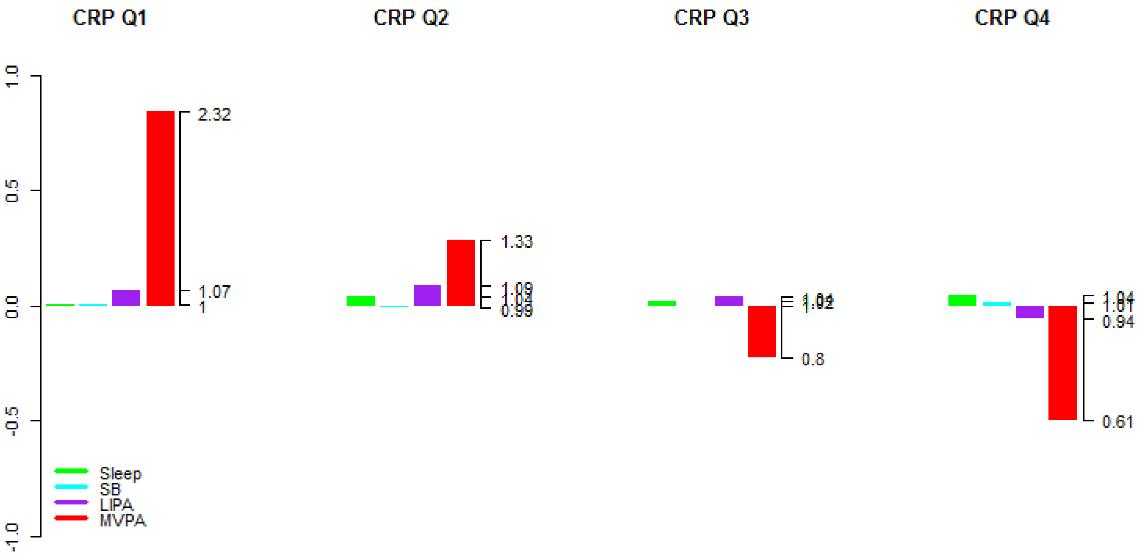

Figure S19. Average time-use compositions of the 24 hour day by CRP quartiles for older adults aged (65-79) in log-ratio scale and relative to the overall average time-use composition at the zero baseline. The left axis gives the log-ratio value and the right axis gives the actual proportion relative to the mean composition (e.g. 1.20 means 1.20 times).

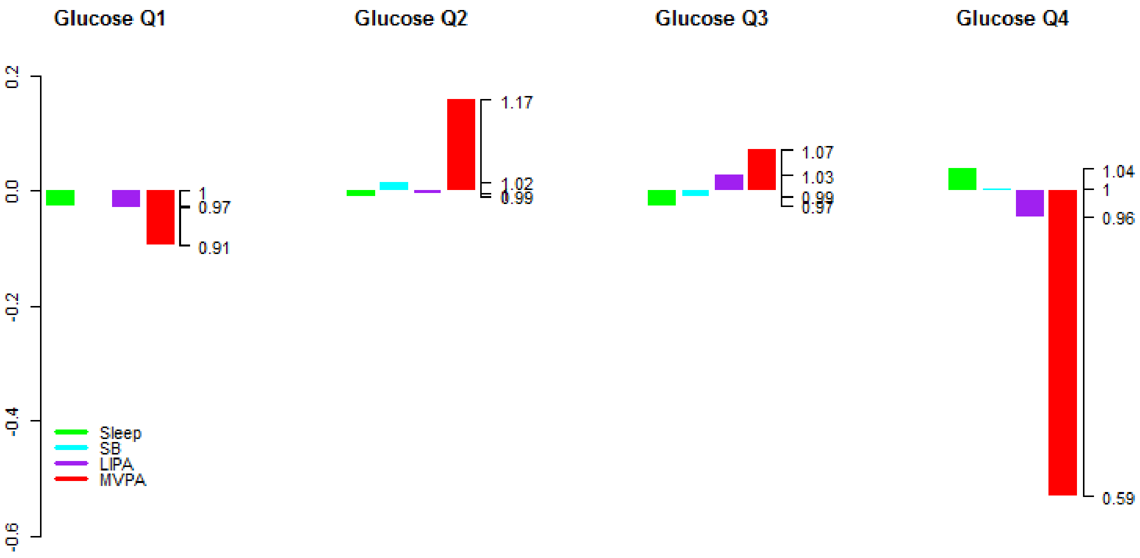

Figure S20. Average time-use compositions of the 24 hour day by blood glucose quartiles for older adults aged (65-79) in log-ratio scale and relative to the overall average time-use composition at the zero baseline. The left axis gives the log-ratio value and the right axis gives the actual proportion relative to the mean composition (e.g. 1.20 means 1.20 times).

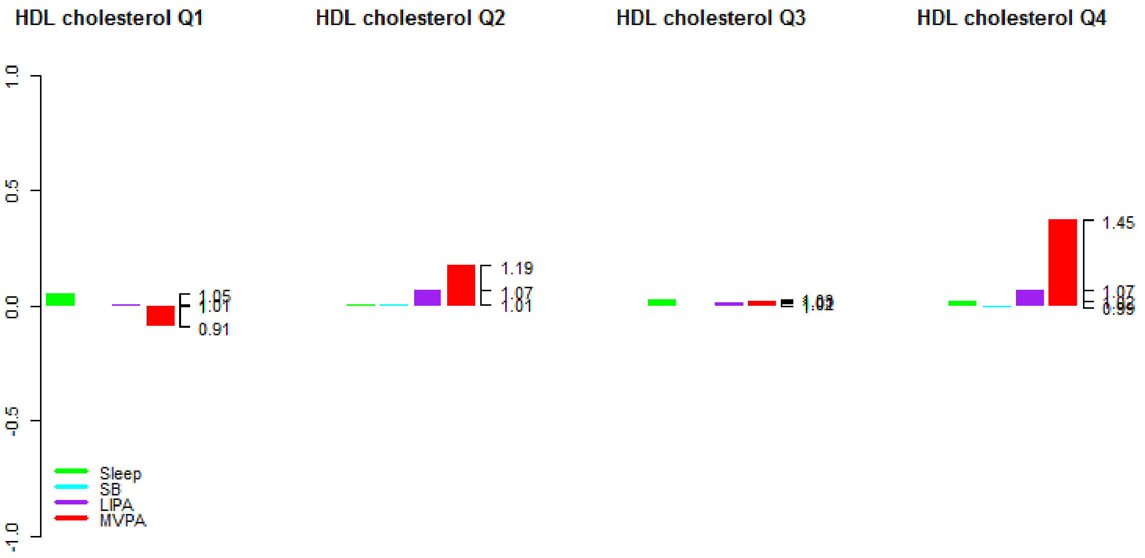

Figure S21. Average time-use compositions of the 24 hour day by HDL cholesterol quartiles for older adults aged (65-79) in log-ratio scale and relative to the overall average time-use composition at the zero baseline. The left axis gives the log-ratio value and the right axis gives the actual proportion relative to the mean composition (e.g. 1.20 means 1.20 times).

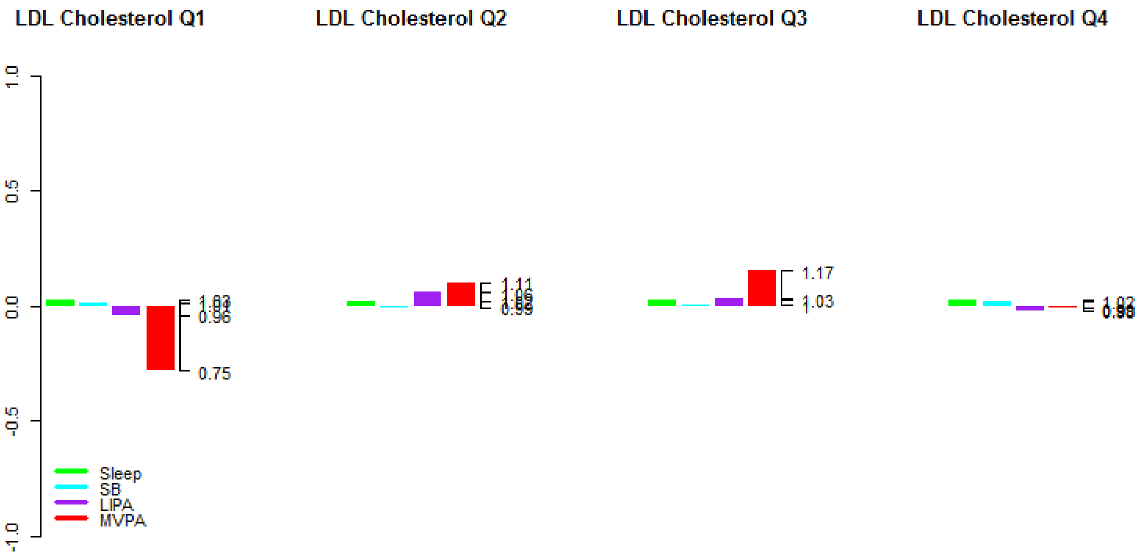

Figure S22. Average time-use compositions of the 24 hour day by LDL cholesterol quartiles for older adults aged (65-79) in log-ratio scale and relative to the overall average time-use composition at the zero baseline. The left axis gives the log-ratio value and the right axis gives the actual proportion relative to the mean composition (e.g. 1.20 means 1.20 times).

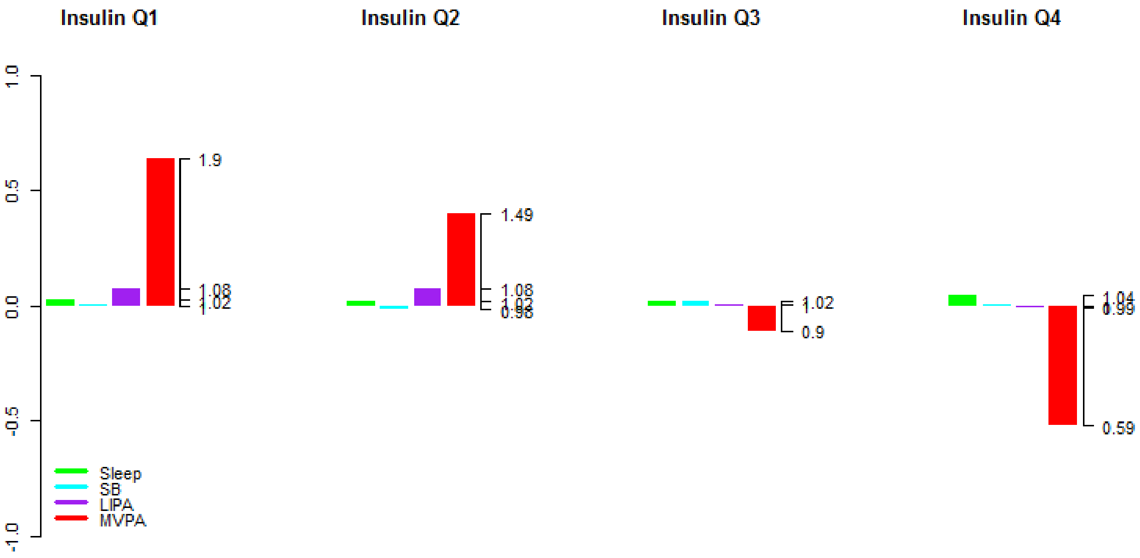

Figure S23. Average time-use compositions of the 24 hour day by insulin quartiles for older adults aged (65-79) in log-ratio scale and relative to the overall average time-use composition at the zero baseline. The left axis gives the log-ratio value and the right axis gives the actual proportion relative to the mean composition (e.g. 1.20 means 1.20 times).

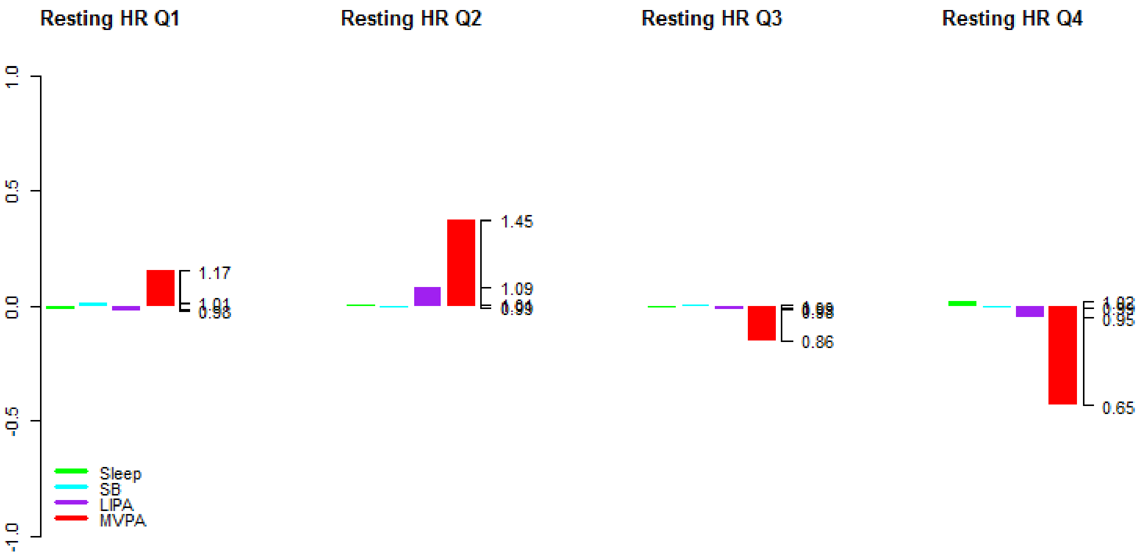

Figure S24. Average time-use compositions of the 24 hour day by resting heart rate quartiles for older adults aged (65-79) in log-ratio scale and relative to the overall average time-use composition at the zero baseline. The left axis gives the log-ratio value and the right axis gives the actual proportion relative to the mean composition (e.g. 1.20 means 1.20 times).

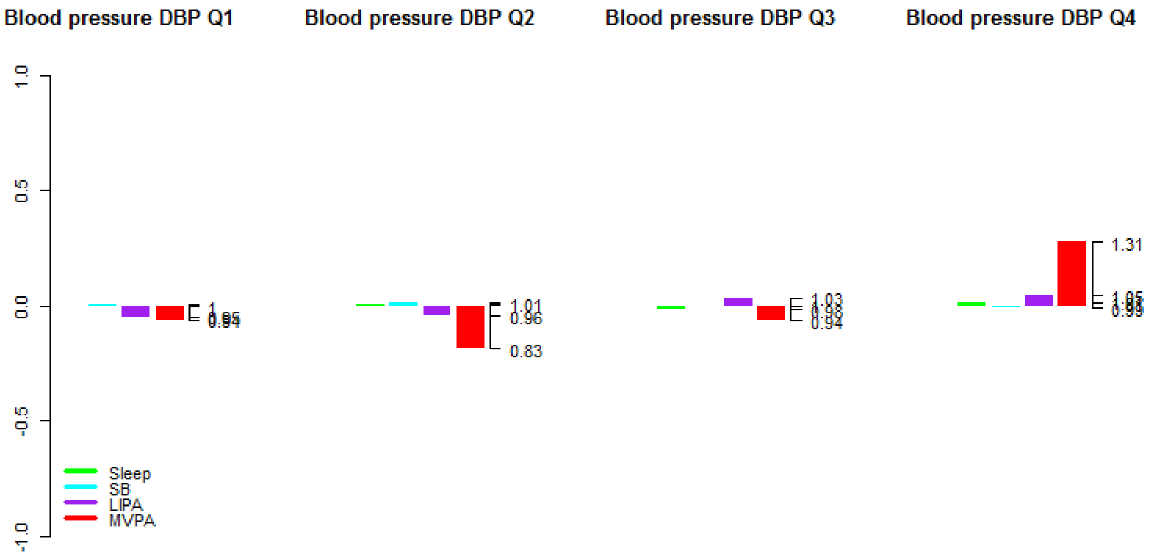

Figure S25. Average time-use compositions of the 24 hour day by diastolic blood pressure quartiles for older adults aged (65-79) in log-ratio scale and relative to the overall average time-use composition at the zero baseline. The left axis gives the log-ratio value and the right axis gives the actual proportion relative to the mean composition (e.g. 1.20 means 1.20 times).

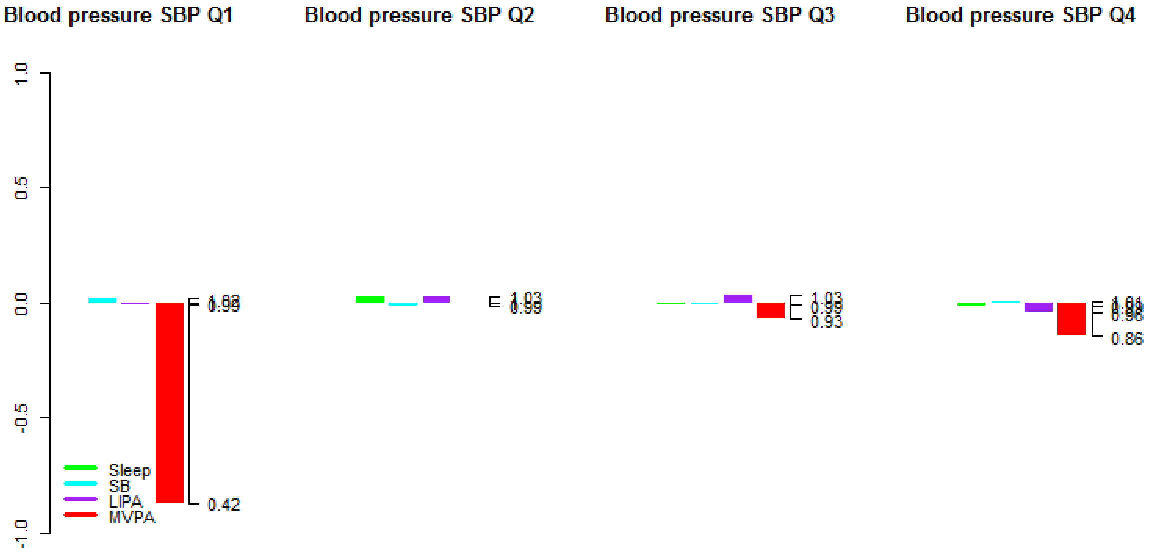

Figure S26. Average time-use compositions of the 24 hour day by systolic blood pressure quartiles for older adults aged (65-79) in log-ratio scale and relative to the overall average time-use composition at the zero baseline. The left axis gives the log-ratio value and the right axis gives the actual proportion relative to the mean composition (e.g. 1.20 means 1.20 times).

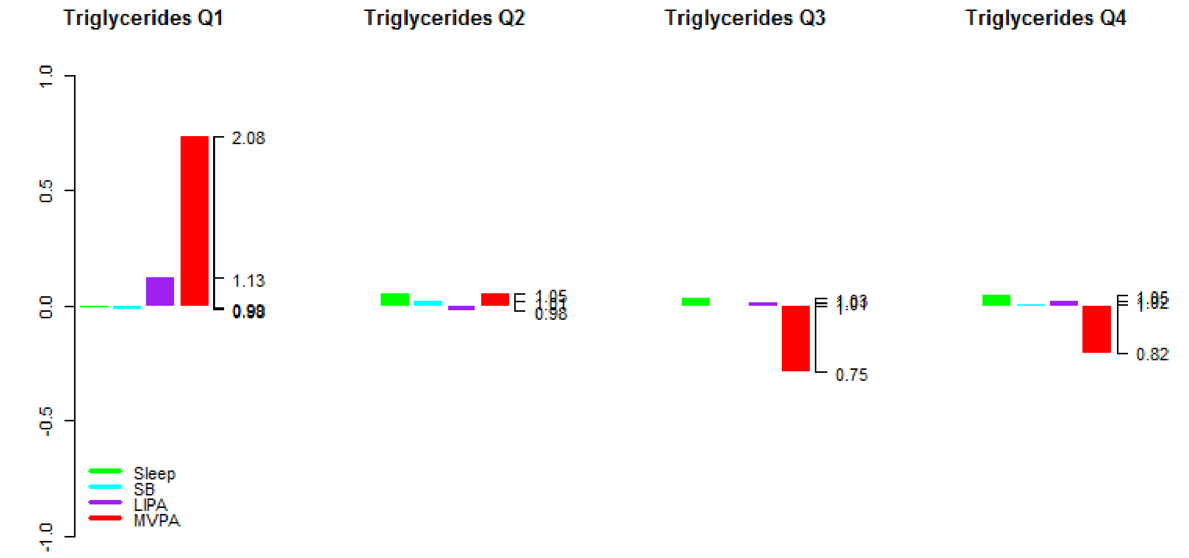

Figure S27. Average time-use compositions of the 24 hour day by triglycerides quartiles for older adults aged (65-79) in log-ratio scale and relative to the overall average time-use composition at the zero baseline. The left axis gives the log-ratio value and the right axis gives the actual proportion relative to the mean composition (e.g. 1.20 means 1.20 times).

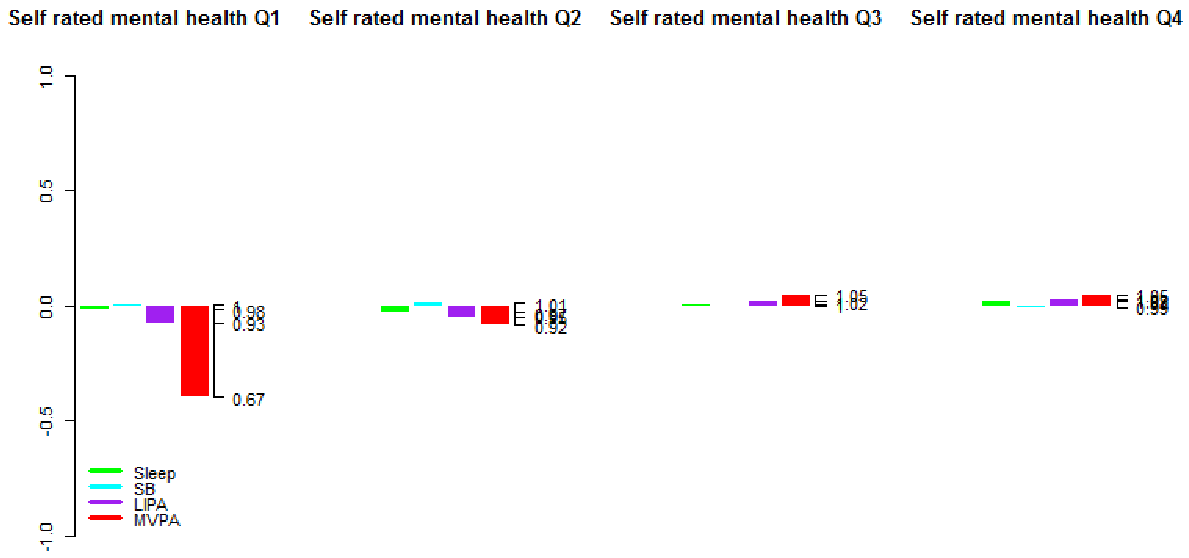

Figure S28. Average time-use compositions of the 24 hour day by self-rated mental health quartiles for older adults aged (65-79) in log-ratio scale and relative to the overall average time-use composition at the zero baseline. The left axis gives the log-ratio value and the right axis gives the actual proportion relative to the mean composition (e.g. 1.20 means 1.20 times).

© 2018 by the authors. Submitted for possible open access publication under the terms and conditions of the Creative Commons Attribution (CC BY) license (http://creativecommons.org/licenses/by/4.0/).
